# How well do frozen foundation models transfer? A calibration-focused benchmark for diabetic retinopathy grading

**DOI:** 10.3389/fmed.2026.1815982

**Published:** 2026-04-16

**Authors:** Mehmet Poyrazer, Hüseyin Yağcı, Rıdvan Erten

**Affiliations:** 1 Division of Endocrinology and Metabolism, University of Health Sciences Ankara Training and Research Hospital, Ankara, Türkiye; 2 Division of Geriatrics, Department of Internal Medicine, Faculty of Medicine, Kocaeli University, Kocaeli, Türkiye

**Keywords:** calibration, diabetic retinopathy, domain shift, external validation, foundation models, frozen encoder, representation transfer, temperature scaling

## Abstract

**Background:**

Pretrained foundation models are increasingly adopted for diabetic retinopathy (DR) screening, yet it remains unclear how much of their performance derives from the learned representations versus the adaptation procedure. Most benchmarks report discrimination metrics alone, neglecting probability calibration.

**Methods:**

We compared the frozen representations of three pretrained encoders: MedSigLIP (medical vision–language; ViT-B/16, 448 × 448), RETFound (retinal self-supervised; ViT-L/16, 224 × 224), and EfficientNet-B0 (ImageNet-supervised; 224 × 224). All encoder weights were frozen; only an identical lightweight multilayer perceptron head was trained. Models were developed on APTOS 2019 (3,662 fundus images; five-fold cross-validation) and externally validated on MESSIDOR-2 (1,744 images). Binary referable DR detection and five-class severity grading were evaluated. AUC, expected calibration error (ECE), and Brier score served as co-primary endpoints. External-set tests used patient-level cluster-robust bootstrap to account for bilateral correlation.

**Results:**

On the development set, all three encoders achieved near-identical binary AUC (0.980–0.985). MedSigLIP showed superior calibration, with a lower Brier score than RETFound (0.044 vs. 0.049; *p* = 0.030) and EfficientNet-B0 (0.044 vs. 0.052; *p* = 0.006). External validation on MESSIDOR-2 revealed divergence: MedSigLIP maintained an AUC of 0.915 (drop 0.070), whereas RETFound fell to 0.697 (drop 0.286) and EfficientNet-B0 to 0.745 (drop 0.236). Retina-specific RETFound performed below the ImageNet baseline (ΔAUC = −0.051; *p* = 0.016, cluster-robust bootstrap). For five-class grading, MedSigLIP achieved an external macro-F1 of 0.450 versus 0.247 (RETFound) and 0.291 (EfficientNet-B0). Temperature scaling reduced development ECE to 0.014–0.022 but proved ineffective under domain shift (external ECE 0.086–0.149). All encoders exhibited catastrophic failure on mild DR (grade 1) externally, with RETFound and EfficientNet-B0 achieving F1 = 0.000 and MedSigLIP only 0.153.

**Conclusion:**

Under frozen transfer, the MedSigLIP encoder package produced more generalisable and better calibrated representations than both retinal self-supervised (RETFound) and ImageNet-supervised (EfficientNet-B0) encoders. Domain-specific pretraining did not guarantee domain-general frozen representations. These findings demonstrate that development-set discrimination alone is insufficient for encoder evaluation and that calibration metrics—particularly the Brier score—should be reported as standard practice.

## 1 Introduction

Diabetic retinopathy (DR) remains the leading cause of preventable blindness among working-age adults worldwide, affecting an estimated 103 million individuals globally, a figure projected to reach 161 million by 2045 (1). Early detection through systematic screening can reduce the risk of severe vision loss by more than 90%, yet the current capacity of trained graders and ophthalmologists falls far short of meeting the growing demand imposed by the global diabetes epidemic (2). Automated analysis of colour fundus photographs using deep learning has therefore attracted sustained interest as a scalable complement to human grading (3, 4). Landmark studies demonstrated that convolutional neural networks can match or exceed the sensitivity and specificity of board-certified ophthalmologists for referable DR detection (3, 5), and at least one system has received regulatory clearance for autonomous screening in primary care (6).

More recently, the field has shifted from training task-specific classifiers towards leveraging pretrained foundation models whose representations can be adapted to downstream clinical tasks with substantially less labelled data (7, 8). In ophthalmology, this paradigm has taken two broadly distinct forms. Domain-specific self-supervised models, exemplified by RETFound, learn transferable features from large corpora of unlabelled retinal images via masked autoencoders (9). Medical vision–language models, such as MedSigLIP, instead align image and text representations across diverse medical modalities using contrastive objectives (10, 11). A third, simpler strategy—using general-purpose ImageNet-pretrained encoders such as EfficientNet—remains a widely deployed baseline owing to its low computational cost and broad availability (12). Each paradigm embeds different inductive biases: retinal self-supervised models favour local structural features; vision–language models encode cross-modal semantic priors; and ImageNet backbones carry strong low-level texture representations. Which of these biases best serves DR classification under domain shift is an open empirical question.

The dominant evaluation methodology in the literature involves full fine-tuning or parameter-efficient adaptation (e.g., LoRA), which confounds two distinct sources of performance: the quality of the pretrained representations themselves and the capacity of the adaptation procedure to reshape them (9, 13). A complementary approach—freezing the encoder and training only a lightweight classification head—isolates the former by eliminating the latter. This frozen-encoder protocol provides a cleaner assay of what the pretrained features already encode about the target task, independent of how much further tuning could improve them. Yet most published comparisons in ophthalmic AI either fine-tune all parameters or evaluate only a single foundation model against conventional baselines, without controlling for adaptation architecture or calibration quality.

A second and arguably more consequential gap concerns calibration. In clinical screening workflows, the confidence that a model assigns to its predictions is as important as their correctness: well-calibrated probability estimates enable threshold-based triage, where high-confidence cases are routed to automated pathways and uncertain cases are escalated for specialist review (14). Modern neural networks are known to be poorly calibrated, often assigning high confidence to incorrect predictions (15). Post-hoc calibration methods such as temperature scaling can mitigate this on in-distribution data, but their effectiveness under domain shift—the very scenario encountered when a model trained on one population is deployed on another—has been insufficiently studied in the DR context. Existing encoder benchmarks rarely report calibration metrics (expected calibration error, Brier score) alongside discrimination metrics (area under the ROC curve), and even fewer evaluate whether calibration transfers across datasets.

To our knowledge, no prior study has systematically compared medical vision–language, domain-specific self-supervised, and general-purpose pretrained encoders for DR classification under an identical frozen-encoder protocol with calibration as a co-primary endpoint and external validation on a geographically distinct cohort. The present work addresses this gap. We benchmark three encoders—MedSigLIP (10), RETFound (9), and EfficientNet-B0 (12)—on two publicly available fundus datasets: APTOS 2019 (16) for model development via five-fold cross-validation and MESSIDOR-2 (17, 18) for zero-tuning external validation. We evaluate both binary referable DR detection (the primary, clinically actionable task) and five-class severity grading according to the International Clinical Diabetic Retinopathy scale (ICDR) (19).

The specific contributions of this study are threefold. First, we provide a controlled comparison of three pretrained encoders spanning distinct pretraining paradigms, with calibration treated as a co-primary endpoint alongside discrimination. Second, we report a transparent contamination audit that maps each dataset to each encoder’s documented pretraining sources, enabling readers to interpret results in light of potential data leakage. Third, we quantify the external generalization gap—the performance drop from APTOS (India, mixed cameras) to MESSIDOR-2 (France, Topcon TRC NW6)—for discrimination, calibration, and ordinal grading metrics, and demonstrate that this gap varies dramatically across encoder paradigms.

## 2 Materials and methods

**Figure 1:**
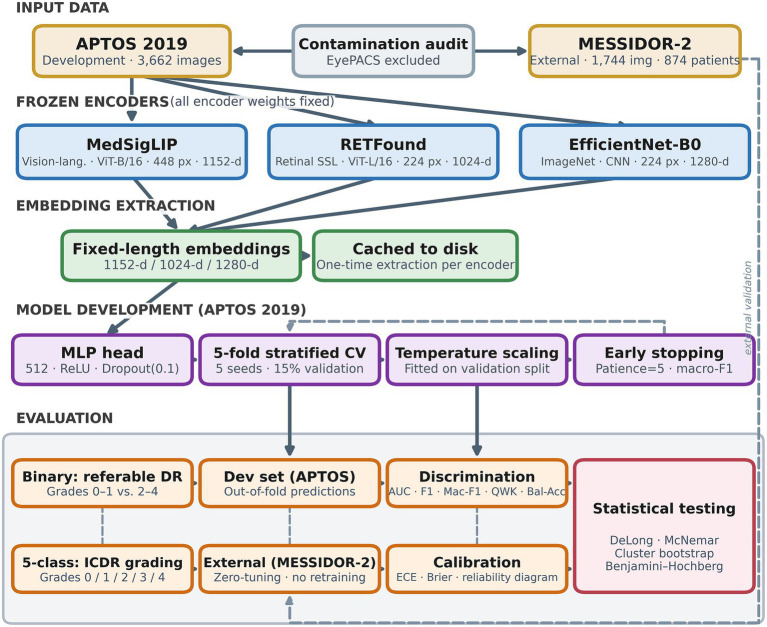
Study workflow. APTOS 2019 (development; *n* = 3,662) and MESSIDOR-2 (external; *n* = 1,744; 874 patients) fundus images serve as input, with EyePACS excluded after contamination audit. Three frozen encoders—MedSigLIP (ViT-B/16; 448 px; 1,152-d), RETFound (ViT-L/16; 224 px; 1,024-d), and EfficientNet-B0 (CNN; 224 px; 1,280-d)—extract fixed-length embeddings cached to disk. A lightweight MLP head (512 units; ReLU; dropout = 0.1) is trained via five-fold stratified cross-validation (five seeds per fold) with early stopping and temperature scaling fitted on the validation split. Trained heads are applied without retraining to MESSIDOR-2. Evaluation covers binary referable DR detection and five-class ICDR grading, assessed by discrimination (AUC, F1, macro-F1, QWK, balanced accuracy), calibration (ECE, Brier score, reliability diagrams), and statistical testing (DeLong, McNemar, cluster bootstrap, Benjamini–Hochberg correction). DR, diabetic retinopathy; ICDR, International Clinical Diabetic Retinopathy; MLP, multilayer perceptron; ECE, expected calibration error; QWK, quadratic weighted kappa.

The study workflow is summarised in Figure 1. Three frozen pretrained encoders extract fixed-length embedding vectors from colour fundus photographs; these vectors are cached to disk and used to train lightweight classification heads via stratified five-fold cross-validation on the APTOS 2019 development set. Trained heads are then applied, without any retraining, to the MESSIDOR-2 external test set. Both binary referable DR detection and five-class ICDR severity grading are evaluated. Temperature scaling is fitted on the validation split of each fold and applied to test and external predictions prior to metric computation.

### 2.1 Datasets

#### 2.1.1 APTOS 2019 (development set)

The APTOS 2019 Blindness Detection dataset (16) comprises 3,662 colour fundus photographs collected at Aravind Eye Hospital, India, using multiple camera systems under variable imaging conditions. Each image carries a severity label on the five-level ICDR scale (19): no DR (grade 0; *n* = 1,805; 49.3%), mild non-proliferative DR (grade 1; *n* = 370; 10.1%), moderate NPDR (grade 2; *n* = 999; 27.3%), severe NPDR (grade 3; *n* = 193; 5.3%), and proliferative DR (grade 4; *n* = 295; 8.1%). For the primary binary task, grades 0–1 were pooled as non-referable (*n* = 2,175; 59.4%) and grades 2–4 as referable (*n* = 1,487; 40.6%). APTOS served as the sole source for model training and internal validation; no images from this dataset were used in any encoder’s pretraining (Table 1, Panel B).

**Table 1:** Dataset characteristics and contamination audit.

Panel A. Distribution of DR severity grades and binary referability labels									
Dataset	Role	*N*	Grade 0	Grade 1	Grade 2	Grade 3	Grade 4	Non-ref.	Referable
APTOS 2019	Development	3,662	1,805 (49.3)	370 (10.1)	999 (27.3)	193 (5.3)	295 (8.1)	2,175 (59.4)	1,487 (40.6)
MESSIDOR-2	External	1,744	1,017 (58.3)	270 (15.5)	347 (19.9)	75 (4.3)	35 (2.0)	1,287 (73.8)	457 (26.2)

Panel B. Contamination audit matrix				
Dataset	Role	MedSigLIP	RETFound	EfficientNet-B0
APTOS 2019	Development	Not confirmed	Evaluation only	No
MESSIDOR-2	External	Not confirmed	Evaluation only	No
EyePACS	EXCLUDED	Pretraining	Pretraining	No
ImageNet-1 K	N/A	No	No	Pretraining

Values are *n* (%). Non-ref., grades 0–1; Referable, grades 2–4. Four ungradable MESSIDOR-2 images excluded. “Not confirmed” = not documented in published pretraining sources. “Evaluation only” = used for downstream evaluation but not pretraining.

#### 2.1.2 MESSIDOR-2 (external test set)

MESSIDOR-2 (17) is an extension of the original MESSIDOR programme and contains 1,748 macula-centred fundus images (874 examinations, two eyes per subject) acquired with a Topcon TRC NW6 non-mydriatic camera at a 45° field of view at Brest University Hospital, France. Because each examination contributes two non-independent images (one per eye), a patient-level identifier was derived from the official left–right eye pairing file to account for bilateral correlation in all external-set statistical analyses (see Section 2.7). The within-patient grade correlation was substantial (Pearson *r* = 0.84; binary concordance 90.9%), confirming that an image-level independence assumption would inflate significance. ICDR severity grades were assigned by the three-expert adjudicated panel described by Krause et al. (18); this consensus grading is widely regarded as the highest-quality reference standard among publicly available DR datasets. Four images flagged as ungradable were excluded, yielding a final cohort of 1,744 images. The referable prevalence in MESSIDOR-2 (26.2%) is substantially lower than in APTOS (40.6%), and the demographic, geographic, and imaging characteristics differ markedly—together creating a stringent test of cross-domain generalization. No images from MESSIDOR-2 were used during model development. RETFound used MESSIDOR-2 for downstream evaluation only, not for self-supervised pretraining (9).

#### 2.1.3 Contamination audit

A pretraining overlap audit was conducted for every candidate dataset–encoder pair (Table 1, Panel B). EyePACS, which appears in the documented pretraining pipelines of both MedSigLIP and RETFound, was excluded from all analyses. FLAIR (Foundation Language-Aligned Image Representations of the Retina) was likewise excluded because its pretraining corpus includes APTOS, IDRiD, and OIA-DDR, which would compromise the fairness of the frozen-encoder comparison. ImageNet-1K, used for EfficientNet-B0 pretraining, contains no ophthalmic imagery. For MedSigLIP, APTOS and MESSIDOR-2 do not appear in the documented pretraining sources, although completeness of disclosure cannot be guaranteed given the partially proprietary nature of the training data (10). We therefore adopt a “contamination-aware” framing throughout: we provide the audit table to enable informed interpretation but make no claim of absolute contamination freedom.

### 2.2 Pretrained encoders

Three encoders were selected to represent three fundamentally different pretraining paradigms, creating a triangular comparison across medical vision–language, retinal self-supervised, and general-purpose strategies.

#### 2.2.1 MedSigLIP (medical vision–language)

MedSigLIP is a medical adaptation of the SigLIP-400M architecture (11), fine-tuned on over 33 million de-identified medical image–text pairs spanning chest radiography, dermatology, ophthalmology, histopathology, and cross-sectional imaging (10). The vision encoder is a Vision Transformer (ViT-B/16) (20) that produces a 1,152-dimensional embedding per image at a native input resolution of 448 × 448 pixels. The sigmoid contrastive loss aligns image and text representations in a shared embedding space, endowing the encoder with cross-modal semantic priors that extend beyond any single imaging modality. In this benchmark, only the vision encoder branch is used; the text encoder is discarded.

#### 2.2.2 RETFound (retinal self-supervised)

RETFound (9) is a ViT-Large/16 foundation model pretrained on approximately 1.6 million colour fundus photographs and optical coherence tomography images from the Moorfields Eye Hospital dataset via masked autoencoder (MAE) self-supervised learning (21). The encoder produces a 1,024-dimensional embedding at 224 × 224 input resolution. As a domain-specific model, RETFound encodes structural priors for retinal anatomy and pathology learned entirely from unlabelled ophthalmic images, without any textual supervision.

#### 2.2.3 EfficientNet-B0 (general-purpose baseline)

EfficientNet-B0 (12) is a compound-scaled convolutional neural network pretrained on ImageNet-1K (22) via standard supervised classification. It produces a 1,280-dimensional embedding at 224 × 224 input resolution. With only 4 million parameters, it serves as a lightweight, general-purpose baseline. Its inclusion enables assessment of whether domain-specific or medical pretraining provides a measurable advantage over transfer from natural images.

Each encoder’s native preprocessing pipeline was preserved: MedSigLIP images were resized to 448 × 448 pixels and normalised with the MedSigLIP-specific processor; RETFound and EfficientNet-B0 images were resized to 224 × 224 pixels and normalised with ImageNet channel means and standard deviations. Resolution differences are an intrinsic property of the pretrained encoders and are explicitly documented as a design choice. However, this disparity means that MedSigLIP receives four times as many input pixels, which may introduce a benchmarking confound; comparisons should therefore be interpreted with this caveat in mind (see Section 4.8 for a detailed discussion).

### 2.3 Classification head architecture

All encoder weights were completely frozen during training. Only a lightweight multilayer perceptron (MLP) head was trained, ensuring that any performance differences reflect the information content of the frozen embeddings rather than optimisation dynamics. The head architecture was identical across all three encoders: a single hidden layer of 512 units with rectified linear unit (ReLU) activation, dropout (*p* = 0.1), and a linear output layer projecting to the number of classes (2 for binary, 5 for five-class).

Training used weighted cross-entropy loss with inverse-frequency class weights computed per fold to mitigate class imbalance. Optimisation was performed with AdamW (23) (learning rate = 1 × 10^−3^, weight decay = 0.01) and a cosine annealing learning rate schedule. The batch size was 64, and training was run for a maximum of 30 epochs with early stopping (patience = 5 epochs, monitoring validation macro-F1). All embeddings were extracted once per encoder and cached to disk prior to head training, guaranteeing that every classifier training run received identical input features. Importantly, this study intentionally evaluates frozen transfer only; results reflect the information content of pretrained embeddings in their unmodified state and should not be extrapolated to fine-tuned performance, which constitutes a separate and complementary evaluation axis (see Section 4.2). To assess the sensitivity of results to head architecture, an ablation study additionally evaluated a single linear layer (logistic regression on frozen embeddings) trained with the same protocol across all three encoders (see Section 4.8).

### 2.4 Cross-validation protocol

Five-fold stratified cross-validation was performed on APTOS 2019. Stratification was applied on the binary label for the primary task and on the five-class grade for the secondary task, preserving class proportions across folds. Within each training fold, 15% of the training data was held out as an internal validation split for early stopping and temperature scaling parameter estimation. For each fold, five independent training runs with different random seeds (seeds: 13, 17, 23, 29, 31) were conducted to quantify MLP initialisation variance. Predictions from all five seeds were soft-averaged (probability ensemble) within each fold before metric computation; no single seed was selected. Seed-level (non-ensembled) metrics are reported in Supplementary Table S1 to demonstrate that within-fold seed variance was negligible (binary AUC SD < 0.002; multiclass macro-F1 SD < 0.021). Out-of-fold predictions on the APTOS test partitions were concatenated across all five folds to yield a single set of development-set predictions for each encoder. As a sensitivity analysis addressing the referability threshold, the binary task was additionally run with an alternative definition (grade ≥ 1 = referable; Supplementary Table S2).

### 2.5 External validation

All five fold-specific MLP checkpoints for each encoder were applied to the full MESSIDOR-2 cohort without any retraining or threshold recalibration. Two reporting strategies were employed. The primary analysis reports fold-wise mean ± SD across the five folds, providing both a point estimate and an uncertainty measure that reflects the variability introduced by different training partitions. A supplementary fold-ensemble analysis (Supplementary Table S3) averages the soft-probability predictions from all five folds before thresholding, representing the practical deployment scenario in which all available heads are combined.

### 2.6 Calibration protocol

Calibration was treated as a co-primary endpoint alongside discrimination, following the rationale that in DR screening programmes, the reliability of a model’s confidence estimates directly governs threshold-based triage decisions.

#### 2.6.1 Temperature scaling

Temperature scaling (15) is a single-parameter post-hoc calibration method. A scalar temperature T is learned by minimising the negative log-likelihood on the validation set; at inference, logits are divided by T before the softmax operation. Crucially, temperature scaling preserves the rank ordering of predictions (and hence AUC) while adjusting only the sharpness of the probability distribution. In each CV fold, T was fitted on the internal validation split of APTOS. For MESSIDOR-2, the same T values were applied directly—no re-tuning was performed on external data—to test whether calibration learned on the development distribution transfers to a new domain.

#### 2.6.2 Calibration metrics

**Figure 2:**
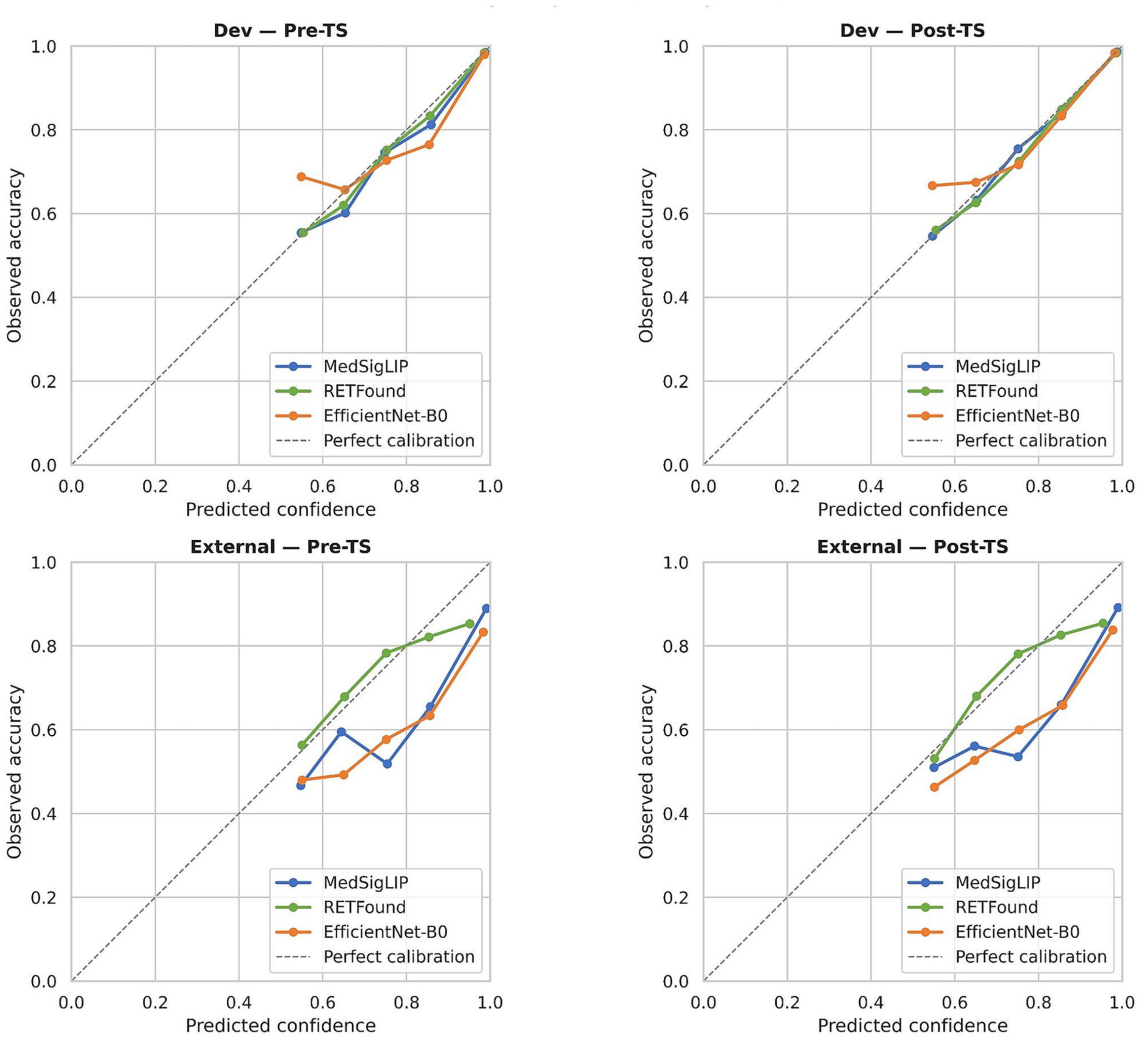
Reliability diagrams for binary referable DR classification. Predicted confidence (horizontal axis) is plotted against observed accuracy (vertical axis) over 10 equal-width bins. Upper row: development set before (left) and after (right) temperature scaling (TS). Lower row: external set before and after TS. The dashed diagonal represents perfect calibration.

Expected calibration error (ECE) was computed using 10 equal-width confidence bins and measures the average absolute gap between predicted confidence and observed accuracy within each bin. The Brier score (24) is the mean squared difference between predicted probabilities and binary outcomes; as a proper scoring rule, it jointly penalises miscalibration and poor discrimination. Reliability diagrams (Figure 2) provide a complementary visual assessment by plotting observed accuracy against predicted confidence per bin, with the identity diagonal representing perfect calibration. All calibration metrics are reported both before and after temperature scaling on both datasets.

### 2.7 Statistical analysis

All pairwise comparisons were conducted among three encoder pairs: MedSigLIP versus RETFound, MedSigLIP versus EfficientNet-B0, and RETFound versus EfficientNet-B0.

For binary AUC, the DeLong test (25) was used, which provides a non-parametric comparison of two correlated receiver operating characteristic curves evaluated on the same sample. Classification correctness was compared using the exact McNemar test (or the continuity-corrected *χ*^2^ variant when the number of discordant pairs exceeded 25) (26). For all remaining metrics—macro-F1, quadratic weighted kappa (QWK) (27), balanced accuracy, ECE, and Brier score—paired bootstrap resampling (1,000 iterations) was employed (28). Bootstrap confidence intervals (95%) for the metric difference (Δ) are reported alongside the corresponding *p*-value.

All *p*-values were corrected for multiple testing within each dataset–task–test-statistic group using the Benjamini–Hochberg procedure (29) to control the false discovery rate at *α* = 0.05. Results are reported as significant only after this correction. Because MESSIDOR-2 contains two eyes per patient (874 patients, 1,744 gradable images), standard image-level resampling would underestimate variance. All external-set bootstrap comparisons were therefore conducted using patient-level cluster resampling (2,000 iterations): at each iteration, 874 patients were drawn with replacement, and all images belonging to each sampled patient were included. This approach yields design-effect–adjusted confidence intervals (estimated design effect ≈ 1.84). DeLong AUC tests, which assume independent observations, are reported on the external set with an ‘unadjusted’ label alongside cluster-robust bootstrap confidence intervals for transparency. Development-set tests remain image-level, as APTOS 2019 does not provide patient identifiers. Effect sizes (Δ metric) are presented with their 95% confidence intervals to enable assessment of both statistical significance and clinical relevance. Selected pairwise comparisons appear in Table 2; the complete set of 36 comparisons is provided in Supplementary Table S4.

**Table 2:** Selected pairwise comparisons.

Set	Comparison	Metric	Test	Δ	95% CI	*p* (BH)
Dev	MedSigLIP vs. RETFound	AUC	DeLong	+0.001	—	0.281
Dev	MedSigLIP vs. RETFound	Brier	Boot	−0.005	[−0.009, −0.001]	0.030^†^
Dev	MedSigLIP vs. RETFound	Mac-F1	Boot	+0.037	[+0.014, +0.059]	0.002^†^
Dev	MedSigLIP vs. EffNet-B0	AUC	DeLong	+0.005	—	0.003^†^
Dev	MedSigLIP vs. EffNet-B0	Brier	Boot	−0.009	[−0.012, −0.004]	0.006^†^
Dev	MedSigLIP vs. EffNet-B0	Mac-F1	Boot	+0.063	[+0.041, +0.086]	0.002^†^
Dev	RETFound vs. EffNet-B0	AUC	DeLong	+0.003	—	0.042^†^
Dev	RETFound vs. EffNet-B0	Mac-F1	Boot	+0.026	[+0.004, +0.048]	0.035^†^
Ext	MedSigLIP vs. RETFound	AUC	Clust. Boot	+0.219	[+0.180, +0.258]	<0.001^†^
Ext	MedSigLIP vs. RETFound	Brier	Clust. Boot	−0.043	[−0.056, −0.030]	0.001^†^
Ext	MedSigLIP vs. RETFound	Mac-F1	Clust. Boot	+0.193	[+0.142, +0.241]	0.001^†^
Ext	MedSigLIP vs. RETFound	QWK	Clust. Boot	+0.365	[+0.307, +0.423]	0.001^†^
Ext	MedSigLIP vs. EffNet-B0	AUC	Clust. Boot	+0.167	[+0.140, +0.196]	<0.001^†^
Ext	MedSigLIP vs. EffNet-B0	Brier	Clust. Boot	−0.053	[−0.064, −0.043]	0.001^†^
Ext	MedSigLIP vs. EffNet-B0	QWK	Clust. Boot	+0.366	[+0.308, +0.423]	0.001^†^
Ext	RETFound vs. EffNet-B0	AUC	Clust. Boot	−0.051	[−0.091, −0.013]	0.016^†^
Ext	RETFound vs. EffNet-B0	QWK	Clust. Boot	+0.001	[−0.061, +0.069]	0.969

Δ = model A minus model B. ^†^Significant at *p* < 0.05 after BH correction. Boot, paired bootstrap (1,000 iterations, image-level). Clust. Boot, patient-level cluster bootstrap (2,000 iterations; used for external set to account for bilateral correlation). CI not applicable (—) for DeLong and McNemar. DeLong AUC tests on the external set (unadjusted for clustering) are reported in Supplementary Table S4.

### 2.8 Software and reproducibility

Experiments were implemented in Python 3.10 using PyTorch 2.1. Encoder weights were loaded from official repositories: MedSigLIP from the Google Health AI Developer Foundations collection on Hugging Face (10), RETFound from the publicly released checkpoint (9), and EfficientNet-B0 from the torchvision model zoo (12). Embedding extraction was performed on a single NVIDIA T4 GPU with mixed-precision inference. Encoder throughput and parameter counts are reported in Supplementary Table S5. The analysis code and configuration files are publicly available, as detailed in the Data Availability Statement.

## 3 Results

### 3.1 Dataset characteristics

Adjudicated gradability labels led to the exclusion of four MESSIDOR-2 images, leaving 3,662 fundus photographs from APTOS for model development and 1,744 from MESSIDOR-2 for external testing. Class distributions were heavily skewed in both datasets (Table 1, Panel A). Nearly half of APTOS comprised grade 0 (49.3%), and this proportion rose to 58.3% in MESSIDOR-2. At the opposite end of the spectrum, severe and proliferative DR accounted for only 13.3% of the development set and a mere 6.3% of the external set. Referable cases—defined here as grades 2 through 4—made up 40.6% of APTOS but only 26.2% of MESSIDOR-2, a gap that, taken alongside the well-documented differences in camera systems and grading protocols between the two cohorts, amounts to a stringent test of cross-domain generalization.

A careful contamination audit confirmed that neither APTOS nor MESSIDOR-2 appeared among the documented pretraining sources for any of the three encoders (Table 1, Panel B). EyePACS—present in both the MedSigLIP and RETFound pretraining pipelines—was excluded from all analyses for this reason. The ImageNet-pretrained EfficientNet-B0 obviously carries no ophthalmic pretraining overlap.

### 3.2 Binary referable DR classification

#### 3.2.1 Development performance

Within the APTOS development set, all three encoders performed well on the referable-versus-non-referable task. MedSigLIP reached a mean AUC of 0.985 ± 0.005 across the five CV folds, while RETFound scored 0.984 ± 0.005 and EfficientNet-B0 0.980 ± 0.007 (Table 3, Panel A). With scores this tightly clustered, it is perhaps unsurprising that the DeLong test detected no statistically significant AUC gap between MedSigLIP and RETFound (Δ = +0.001; *p* = 0.281), although MedSigLIP did edge past EfficientNet-B0 with a small but reproducible margin (Δ = +0.005; *p* = 0.003). Binary F1 scores fell within a similarly narrow band—between 0.917 and 0.928—and none of the McNemar comparisons survived BH correction (Table 2).

**Table 3:** Main benchmark performance (post-temperature scaling).

Panel A. Binary referable DR classification.								
Encoder	Dev AUC	Ext AUC	Dev F1	Ext F1	Dev ECE↓	Ext ECE↓	Dev Brier↓	Ext Brier↓
MedSigLIP	0.985 ± 0.005	0.915 ± 0.005	0.928 ± 0.015	0.606 ± 0.024	0.014 ± 0.006	0.109 ± 0.011	0.044 ± 0.007	0.127 ± 0.006
RETFound	0.984 ± 0.005	0.697 ± 0.009	0.917 ± 0.014	0.443 ± 0.046	0.018 ± 0.007	0.086 ± 0.020	0.049 ± 0.009	0.185 ± 0.024
EfficientNet-B0	0.981 ± 0.007	0.745 ± 0.012	0.916 ± 0.021	0.365 ± 0.059	0.022 ± 0.007	0.149 ± 0.023	0.052 ± 0.011	0.182 ± 0.012

Panel B. Five-class DR severity grading										
Encoder	Dev Mac-F1	Ext Mac-F1	Dev QWK	Ext QWK	Dev Bal-Acc	Ext Bal-Acc	Dev ECE↓	Ext ECE↓	Dev Brier↓	Ext Brier↓
MedSigLIP	0.708 ± 0.018	0.450 ± 0.022	0.906 ± 0.008	0.719 ± 0.014	0.717 ± 0.016	0.420 ± 0.018	0.029 ± 0.014	0.216 ± 0.014	0.232 ± 0.012	0.536 ± 0.018
RETFound	0.670 ± 0.012	0.247 ± 0.029	0.884 ± 0.016	0.331 ± 0.026	0.679 ± 0.018	0.300 ± 0.017	0.039 ± 0.009	0.208 ± 0.016	0.275 ± 0.010	0.673 ± 0.026
EfficientNet-B0	0.644 ± 0.012	0.291 ± 0.022	0.857 ± 0.016	0.360 ± 0.038	0.659 ± 0.012	0.313 ± 0.031	0.032 ± 0.007	0.275 ± 0.020	0.289 ± 0.006	0.678 ± 0.015

Mean ± SD across five stratified CV folds.

AUC, area under the ROC curve; F1, binary positive-class F1; ECE, expected calibration error (10 bins); Brier, Brier score; Mac-F1, macro-averaged F1; QWK, quadratic weighted kappa; Bal-Acc, balanced accuracy. ↓ = lower is better. Bold = best per column. Dev, out-of-fold on APTOS; Ext, zero-tuning on MESSIDOR-2.

Where the three encoders did diverge, even at this near-ceiling level of discrimination, was in the quality of their probability estimates. MedSigLIP’s Brier score on the development set (0.044 ± 0.007) was lower than that of both RETFound (0.049 ± 0.009; Δ = −0.005, 95% CI [−0.009, −0.001]; *p* = 0.030) and EfficientNet-B0 (0.052 ± 0.011; Δ = −0.008, 95% CI [−0.012, −0.004]; *p* = 0.006). The Brier score is a proper scoring rule that penalises both miscalibration and poor discrimination, so this finding suggests that MedSigLIP embeddings supplied a richer probabilistic signal to the downstream MLP than the alternatives did, even when headline AUC figures looked nearly identical.

#### 3.2.2 External validation

**Figure 3:**
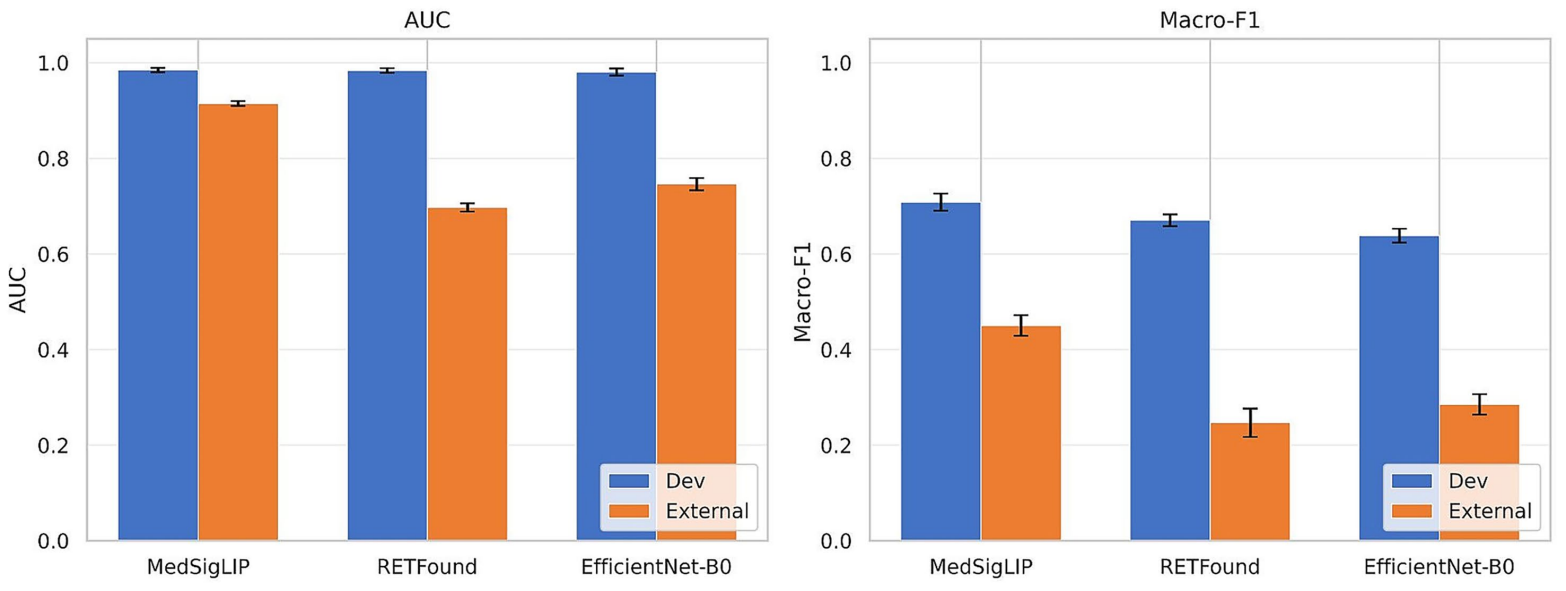
Discriminative performance summary. Left: Binary area under the receiver operating characteristic curve (AUC). Right: Five-class macro-averaged F1 (macro-F1). Blue bars represent the APTOS 2019 development set (out-of-fold); orange bars the MESSIDOR-2 external set (zero-tuning). Bar height = fold-averaged mean; error bars = ± 1 standard deviation (SD). All values are post-temperature-scaling.

Applying the APTOS-trained MLP heads to MESSIDOR-2 without any further tuning exposed stark differences among encoders (Table 3, Panel A; Figure 3, left). MedSigLIP retained a respectable AUC of 0.915 ± 0.005—a drop of only 0.070 from its development-set figure. RETFound, by comparison, fell to 0.697 ± 0.009 (gap: 0.286), and EfficientNet-B0 to 0.745 ± 0.012 (gap: 0.236). Put differently, the generalization deficit for MedSigLIP was three to four times smaller than that of the other encoders.

All six pairwise AUC comparisons on the external set achieved statistical significance under cluster-robust bootstrap resampling that accounts for bilateral correlation within patients (Table 2; complete results in Supplementary Table S4). The gap separating MedSigLIP from RETFound remained striking: ΔAUC = 0.219 (95% CI [0.180, 0.258], cluster-robust *p* < 0.001); the MedSigLIP versus EfficientNet-B0 gap was similarly robust (ΔAUC = 0.167, 95% CI [0.140, 0.196], *p* < 0.001). Equally noteworthy, MedSigLIP exceeded EfficientNet-B0 by 0.167 AUC units (*p* < 0.001). However, a particularly noteworthy observation from the binary analysis is that RETFound—a model pretrained on over 1.6 million colour fundus photographs—performed significantly below the general-purpose ImageNet backbone on this external dataset (ΔAUC = −0.051; *p* = 0.016, cluster-robust). McNemar’s test reinforced these observations at the level of individual classification decisions: MedSigLIP was correct on 7.8 percentage points more images than RETFound (*p* < 0.001) and on 6.5 percentage points more than EfficientNet-B0 (*p* < 0.001).

### 3.3 Five-class severity grading

The transition from binary screening to the full five-class ICDR grading task widened the performance hierarchy established above. On the APTOS development set, MedSigLIP achieved a macro-F1 of 0.708 ± 0.018 and a QWK of 0.906 ± 0.008 (Table 3, Panel B; Figure 3, right). RETFound (macro-F1: 0.670 ± 0.012; QWK: 0.884 ± 0.016) and EfficientNet-B0 (0.644 ± 0.012; 0.857 ± 0.016) both trailed by margins that were statistically significant across every pairwise comparison (all *p* ≤ 0.035; Table 2). Balanced accuracy followed the same ordering: 0.717 for MedSigLIP, 0.679 for RETFound, 0.659 for EfficientNet-B0.

External generalization proved difficult for all encoders, but the relative ranking was preserved and the gaps grew larger. MedSigLIP’s macro-F1 on MESSIDOR-2 (0.450 ± 0.022) more than doubled the scores returned by RETFound (0.247 ± 0.029) and EfficientNet-B0 (0.291 ± 0.022), both comparisons reaching significance (*p* < 0.001, cluster-robust bootstrap; Supplementary Table S4). Its external QWK of 0.719 ± 0.014 falls within what Landis and Koch would classify as “substantial” agreement—in contrast to the “fair” scores of RETFound (0.331 ± 0.026) and EfficientNet-B0 (0.360 ± 0.038). One external comparison was non-significant: the QWK difference between RETFound and EfficientNet-B0 (Δ = +0.001; *p* = 0.969), suggesting that, absent a strong vision–language prior, neither retinal nor general pretraining offered a meaningful advantage in ordinal severity ranking under domain shift.

Five-class calibration metrics, omitted in the original submission, are now reported in Table 3, Panel B. On the development set, all three encoders achieved moderate ECE after temperature scaling (MedSigLIP 0.029 ± 0.014; EfficientNet-B0 0.032 ± 0.007; RETFound 0.039 ± 0.009). Under domain shift, calibration degraded substantially for all encoders, but MedSigLIP (ECE 0.216 ± 0.014; Brier 0.536 ± 0.018) maintained the best multiclass Brier score—significantly lower than both RETFound (Brier 0.673 ± 0.026; Δ = −0.132, *p* < 0.001) and EfficientNet-B0 (Brier 0.678 ± 0.015; Δ = −0.144, *p* < 0.001) under cluster-robust bootstrap. RETFound’s external five-class ECE (0.208 ± 0.016) was slightly lower than MedSigLIP’s, mirroring the binary-task pattern where apparently favourable ECE reflects probability compression rather than genuine reliability (see Section 4.5).

#### 3.3.1 Per-grade analysis

Grade-level breakdown of the external results (Supplementary Table S6) brought an important clinical nuance into focus. Mild NPDR (grade 1)—the category defined by the presence of scattered microaneurysms alone—proved catastrophically difficult. RETFound and EfficientNet-B0 both returned an F1 of exactly 0.000 for this grade, failing to correctly identify a single mild case in the 270 MESSIDOR-2 images carrying that label. MedSigLIP fared somewhat better, but its grade-1 F1 of 0.153 is hardly reassuring. At the severe end, the picture reversed: MedSigLIP classified proliferative DR (grade 4; *n* = 35) with F1 = 0.590, whereas RETFound managed only 0.056 and EfficientNet-B0 0.308.

The confusion matrices (Supplementary Figure S1) clarified the mechanism behind these numbers. Under domain shift, both RETFound and EfficientNet-B0 exhibited a pronounced tendency to assign higher-severity cases to grade 0—effectively defaulting to a “normal” prediction whenever the encoder’s frozen features did not clearly signal pathology. MedSigLIP’s errors were more evenly distributed across the off-diagonal, consistent with a representation that captures finer grading distinctions even if it lacks sufficient domain-specific resolution to exploit them fully. An error overlap analysis (Supplementary Figure S2) confirmed that images correctly classified exclusively by MedSigLIP outnumbered those classified exclusively by either competitor, pointing to features in the MedSigLIP embedding space to which the other two encoders are largely blind.

### 3.4 Calibration and temperature scaling

Before any post-hoc adjustment, all three binary classifiers were already well calibrated on the development set, with expected calibration errors (ECE, 10 bins) of 0.015 for MedSigLIP, 0.021 for RETFound, and 0.024 for EfficientNet-B0 (Figure 2, upper-left). Temperature scaling—fitted on the held-out validation fold—tightened these values only marginally (post-TS ECE: 0.014, 0.018, and 0.022, respectively), which is not unexpected given that the starting calibration was already good. The estimated temperatures were modest: 1.11 ± 0.12 for MedSigLIP, 1.02 ± 0.13 for RETFound, and 1.28 ± 0.13 for EfficientNet-B0. Five-class temperature values, which showed similar patterns with slightly higher fold-to-fold variability, are reported in Supplementary Table S7. It is worth noting that the softmax function imposes a mathematical lower bound on predicted confidence of 1/K (0.50 for binary, 0.20 for five-class), meaning that none of the models can express genuine uncertainty in the form of near-zero confidence; this property is visible in the reliability diagrams (Figure 2), where no predicted confidence falls below approximately 0.5.

The external picture was less encouraging. Reliability diagrams (Figure 2, lower panels) showed systematic overconfidence for every encoder, with predicted probabilities exceeding actual accuracy by roughly 5–15 percentage points across most confidence bins. Post-TS ECE values on MESSIDOR-2 were 0.109 ± 0.011 (MedSigLIP), 0.086 ± 0.020 (RETFound), and 0.149 ± 0.023 (EfficientNet-B0). The seemingly favourable ECE for RETFound here should be interpreted with caution (see Section 4.5): its probability mass is compressed near the decision boundary, which can yield a low ECE arithmetically while the underlying predictions carry poor discriminative content (recall that its AUC was only 0.697). Temperature scaling barely moved the needle on the external set—ECE reductions of 0.004 for MedSigLIP, an increase of 0.002 for RETFound, and a modest reduction of 0.014 for EfficientNet-B0—temperature scaling reduced miscalibration only modestly under cross-domain evaluation and did not fully correct calibration errors, as reflected by remaining ECE/Brier values.

Once again, the Brier score offered the most integrated view of external predictive quality. MedSigLIP’s external Brier score (0.127 ± 0.006) was significantly lower—and hence better—than both RETFound’s (0.185 ± 0.024; Δ = −0.043, 95% CI [−0.056, −0.030]; *p* < 0.001) and EfficientNet-B0’s (0.182 ± 0.012; Δ = −0.053, 95% CI [−0.064, −0.043]; *p* < 0.001); all differences remained significant under patient-level cluster-robust bootstrap (Supplementary Table S4).

### 3.5 Stability across folds and random seeds

Across the five CV folds, the standard deviation of development-set binary AUC was 0.005 for both MedSigLIP and RETFound, and 0.007 for EfficientNet-B0. Seed-to-seed variability was even smaller: within any given fold, repeating the MLP training with five different seeds (seeds: 13, 17, 23, 29, 31) produced AUC fluctuations with an SD below 0.002 (Supplementary Table S1). These numbers suggest that the performance gaps documented above reflect genuine differences in the information content of the frozen embeddings, not artefacts of data splitting or stochastic optimisation. All model–fold–seed configurations converged within 15–25 epochs (Supplementary Figure S3), with no signs of overfitting on the held-out validation split.

## 4 Discussion

### 4.1 Summary of principal findings

This study compared the frozen representations of three pretrained encoders—MedSigLIP, RETFound, and EfficientNet-B0—for diabetic retinopathy classification under a controlled, calibration-focused protocol with external validation. Three principal findings emerged. First, on the APTOS development set, all three encoders achieved near-ceiling binary discrimination (AUC 0.980–0.985), yet MedSigLIP already showed superior probability calibration, as reflected in a significantly lower Brier score (0.044 vs. 0.049–0.052; *p* ≤ 0.030). Second, on the geographically and demographically distinct MESSIDOR-2 external set, MedSigLIP retained an AUC of 0.915 whereas RETFound and EfficientNet-B0 dropped to 0.697 and 0.745, respectively—a three- to four-fold difference in the magnitude of the generalization deficit. Third, temperature scaling, although effective on development data, failed to correct the structured miscalibration introduced by domain shift for any encoder, highlighting a fundamental limitation of single-parameter post-hoc calibration when the source and target distributions diverge.

### 4.2 Frozen-encoder evaluation as a representation assay

The frozen-encoder protocol adopted in this study isolates representation quality from the confounding effects of adaptation capacity. When all parameters are tunable, a sufficiently expressive adaptation procedure can compensate for mediocre pretrained features, obscuring genuine differences in what the encoder has learned. By constraining adaptation to an identical, lightweight MLP head, we ensure that the observed performance hierarchy reflects differences in the information content of the embeddings themselves. This design choice aligns with the linear-probing paradigm widely used in the representation learning literature (30), extended here with a single nonlinear hidden layer to accommodate the higher complexity of fine-grained medical classification.

It is important to note what this study does not claim. Frozen-encoder performance is not a predictor of what each model can achieve under full fine-tuning or parameter-efficient adaptation [e.g., LoRA (13)]. RETFound, for instance, achieved an AUROC of 0.822 on MESSIDOR-2 when fine-tuned on APTOS (9)—the same development–external pair used in the present benchmark—substantially higher than the frozen-transfer AUROC of 0.697 observed here, underscoring that frozen performance is an assay of representational readiness rather than an upper bound on model capability. The frozen protocol tests a different and complementary research question: not how adaptable the representations are, but how informative they are in their pretrained state. Both properties are relevant for clinical deployment—the former when computational resources permit retraining, the latter when models must be applied off-the-shelf or when regulatory constraints limit retraining cycles.

### 4.3 Cross-modal priors and domain-shift resilience

A notable finding of this benchmark is MedSigLIP’s superior external generalization. Its development-to-external AUC drop (Δ = 0.070) was markedly smaller than those of RETFound (Δ = 0.286) and EfficientNet-B0 (Δ = 0.236). We attribute this resilience to the nature of the pretraining signal. MedSigLIP’s contrastive vision–language objective forces the encoder to learn features that are invariant across imaging conditions, anatomical sites, and clinical descriptions (10, 11). This semantic grounding may render the representations less sensitive to the low-level distributional shifts—changes in camera hardware, illumination, field of view, and population demographics—that characterise the APTOS-to-MESSIDOR-2 transition.

RETFound’s comparatively limited external performance was noteworthy. As a model pretrained on 1.6 million retinal images, one might anticipate that its representations would transfer readily across fundus datasets. Yet the frozen RETFound embeddings yielded an external AUC of only 0.697—significantly below the general-purpose ImageNet baseline (ΔAUC = −0.051; *p* = 0.016, cluster-robust bootstrap). This suggests that the MAE objective, while effective at learning structural features for the pretraining domain (UK Biobank, Moorfields Eye Hospital), may produce representations that are tightly coupled to the visual statistics of the training distribution. When these statistics shift—different camera, different patient population, different grading protocol—the frozen features lose discriminative power. Furthermore, this divergence can be largely attributed to the fundamental differences in pretraining objectives. MAEs like RETFound are optimised to reconstruct missing pixels, a task that encourages the model to learn highly localised, entangled features. As established in representation learning literature, MAE embeddings typically require full non-linear fine-tuning to become linearly separable for downstream classification tasks (21). Conversely, contrastive vision-language models like MedSigLIP explicitly align global image representations with semantic text features during pretraining. This objective naturally produces globally linearly separable embeddings that are inherently well-suited for frozen transfer and linear probing methodologies. Therefore, RETFound’s underperformance highlights an architectural mismatch for resource-constrained ‘off-the-shelf’ deployment rather than a lack of domain knowledge. Importantly, this does not diminish the value of RETFound as a fine-tunable foundation model; it highlights, rather, that domain-specificity in pretraining does not necessarily confer domain-generality in frozen transfer.

EfficientNet-B0, despite carrying no medical pretraining, outperformed RETFound on the external set for both binary AUC and five-class macro-F1. This outcome echoes a broader pattern observed across transfer learning benchmarks: ImageNet features encode low-level visual primitives (edges, textures, colour gradients) that, while not domain-specific, are sufficiently general to support many downstream tasks (31). The simplicity of this baseline underscores the importance of including general-purpose models in foundation model evaluations.

### 4.4 Grade-1 classification failure under domain shift

The per-grade analysis revealed that mild NPDR (grade 1) was catastrophically difficult for all encoders on the external set, but the failure modes differed qualitatively. RETFound and EfficientNet-B0 achieved an F1 of exactly 0.000 for this grade, failing to correctly identify a single mild case among 270 MESSIDOR-2 images (Supplementary Table S6). Their confusion matrices (Supplementary Figure S1) showed a systematic tendency to assign higher-severity cases to grade 0, effectively defaulting to a “normal” prediction whenever the frozen features did not clearly signal pathology. MedSigLIP fared modestly better (F1 = 0.153), maintaining non-zero recall (15.2%) and a bimodal confidence distribution (Supplementary Figure S4) that suggests its embeddings capture at least partial information about mild pathological changes.

Mild NPDR is defined by the presence of scattered microaneurysms alone (19), lesions that are small, subtle, and easily confounded with imaging artefacts or normal vascular variation. Under domain shift, the visual signature of these lesions changes with camera resolution, illumination, and fundus pigmentation. The clinical implication is notable: even the best-performing frozen encoder in this benchmark would miss approximately 85% of mild DR cases in an external population. This finding motivates a two-stage screening design in which frozen-encoder triage is combined with specialist review for cases near the referable/non-referable boundary.

It should be noted that classifying grade 1 as non-referable follows the ICDR standard and is the most common convention in automated DR screening studies (3, 4). However, several national programmes (e.g., the UK NHS Diabetic Eye Screening Programme) define any level of retinopathy as screen-positive. A sensitivity analysis using the alternative threshold (grade ≥ 1 = referable) is reported in Supplementary Table S2. Under this definition, MedSigLIP maintained a clear external AUC advantage (0.867 ± 0.012) over both RETFound (0.654 ± 0.007) and EfficientNet-B0 (0.675 ± 0.007), preserving the encoder ranking observed under the primary threshold. However, the domain-shift AUC drop was larger for all encoders (MedSigLIP: Δ = 0.132 vs. 0.070; RETFound: Δ = 0.342 vs. 0.286; EfficientNet-B0: Δ = 0.322 vs. 0.235), consistent with the increased difficulty of detecting subtle grade-1 lesions under distribution shift.

### 4.5 Calibration under domain shift

A distinguishing feature of this benchmark is the treatment of calibration as a co-primary endpoint. On the development set, all three binary classifiers were well calibrated after temperature scaling (ECE 0.014–0.022), consistent with the expectation that post-hoc adjustment can correct moderate overconfidence when the source and calibration distributions match. However, on the external set, temperature scaling barely reduced ECE—and in the case of RETFound, it slightly increased miscalibration. Post-temperature-scaling external ECE values ranged from 0.086 to 0.149 (Figure 2), suggesting that a single scalar temperature may be insufficient to correct structured, bin-level miscalibration under distributional shift (32, 33).

RETFound’s seemingly favourable external ECE (0.086, the lowest of the three) warrants cautious interpretation. Its probability mass is compressed near the decision boundary, which can yield a low ECE arithmetically—many images receive predictions close to 0.5, and accuracy in that confidence range is also close to chance. The Brier score, which integrates both calibration and discrimination, exposes this pattern: RETFound’s external Brier (0.185) is substantially worse than MedSigLIP’s (0.127; Δ = −0.043, *p* < 0.001). We therefore recommend that ECE be interpreted alongside discrimination metrics and the Brier score, rather than in isolation, to avoid misleading conclusions about calibration quality.

The confidence distribution analysis for grade-1 images (Supplementary Figure S4) revealed a particularly concerning pattern: RETFound and EfficientNet-B0 assigned high confidence (0.9–1.0) to their grade-1 predictions despite achieving F1 = 0.000—systematic overconfidence on systematically incorrect classifications. In a clinical triage system that routes high-confidence predictions to automated pathways, such behaviour would result in confidently missed mild DR, the very scenario that calibration-aware evaluation is designed to detect.

More broadly, the softmax activation guarantees a minimum confidence of 1/K (0.50 for binary, 0.20 for five-class), which prevents any model from flagging truly uncertain cases with low confidence scores. In a clinical triage system designed to route uncertain images to specialist review, this property is a fundamental limitation: the models are always decisive, even when they are wrong. Complementary uncertainty quantification methods—such as Monte Carlo dropout, deep ensembles, or conformal prediction—would be needed to produce meaningful abstention signals for deployment in screening workflows.

### 4.6 Error overlap and ensemble potential

The error overlap analysis (Supplementary Figure S2) provides insight into whether the three encoders extract complementary or redundant features. On the MESSIDOR-2 binary task, the largest error bar corresponded to images that all three models classified incorrectly, pointing to a substantial core of systemically difficult cases that likely reflect genuine ambiguity or artefactual degradation. The M1_R0_E0 pattern—MedSigLIP correct, both competitors incorrect—substantially outnumbered the reverse patterns, confirming that MedSigLIP captures visual information to which the other encoders are largely blind. However, the fold-ensemble analysis (Supplementary Table S3) showed that averaging predictions from five folds did not meaningfully rescue external performance for any encoder, suggesting that the dominant source of error is representational (what the encoder cannot see) rather than stochastic (variability across training splits).

### 4.7 Practical considerations

The encoder profiles (Supplementary Table S5) highlight a throughput–performance trade-off. MedSigLIP (878 M parameters) processes 12.5 images per second on an NVIDIA T4 GPU, compared with 106.8 for RETFound (303 M) and 1,211.6 for EfficientNet-B0 (4 M). In resource-constrained screening settings where inference latency and hardware cost are critical, EfficientNet-B0 remains a pragmatic choice—it is 97 × faster than MedSigLIP and achieved reasonable development-set performance. Conversely, for centralised cloud-based screening where throughput is less constrained, MedSigLIP’s markedly superior external generalization and calibration may justify its higher computational cost. These considerations reinforce the broader principle that encoder selection should be guided by the deployment context, not by development-set AUC alone.

### 4.8 Limitations

Several limitations should be acknowledged. First, the frozen-encoder protocol deliberately excludes fine-tuning and parameter-efficient adaptation, which means the results do not reflect the full potential of each model. RETFound, in particular, was designed and validated with full fine-tuning (9), and its poor frozen-transfer performance should not be extrapolated to clinical scenarios where retraining is feasible. Recent work on multi-stage DR severity classification using transfer learning (34), encoder-decoder architectures for diabetic eye disease segmentation (35), and transformer-based approaches for fundus disease detection (36) highlight the growing interest in representation-based approaches, although these studies employ full fine-tuning rather than the frozen-encoder protocol adopted here.

Second, the resolution disparity between MedSigLIP (448 × 448) and the other two encoders (224 × 224) introduces a fundamental confound. MedSigLIP receives four times as many input pixels, which undoubtedly provides a structural advantage in detecting subtle, pixel-level lesions such as the isolated microaneurysms that define mild NPDR (grade 1). However, this disparity is an inherent, hardcoded property of the pretrained Vision Transformer architectures. Forcing an artificial uniform resolution—such as downsampling MedSigLIP to 224 × 224—would distort its pretrained positional embeddings and severely degrade the representation quality, violating the model card specifications. Consequently, evaluating each model at its native, natively engineered resolution is the only methodologically sound approach for an ‘off-the-shelf’ frozen benchmark, even though it precludes a strictly resolution-controlled comparison. This design means that the relative contributions of input resolution and vision–language pretraining to MedSigLIP’s external advantage cannot be fully disentangled, and readers should interpret the reported performance hierarchy with this asymmetry in mind.

Third, APTOS 2019 provides image-level labels without patient-level identifiers, preventing patient-level stratification of the cross-validation folds. If multiple images from the same patient appear in both training and test partitions, development-set performance estimates may be mildly optimistic; however, this concern does not affect the external test set, where no patient overlap with APTOS is possible. For the external set, a separate but related concern arises from MESSIDOR-2’s bilateral structure (two eyes per patient, 874 patients). This within-patient correlation (Pearson *r* = 0.84; binary concordance 90.9%) was addressed through patient-level cluster-robust bootstrap resampling, yielding an estimated design effect of 1.84 (effective *N* ≈ 948). Despite wider confidence intervals, all primary pairwise comparisons retained statistical significance (Table 2), indicating that the conclusions are robust to the clustering structure. Fourth, the contamination audit relies on publicly available documentation of each encoder’s pretraining data. For MedSigLIP, which was trained on a mixture of public and proprietary medical datasets (10), we cannot definitively rule out the presence of APTOS or MESSIDOR-2 images in the proprietary component. We report this uncertainty transparently rather than claiming contamination freedom. Fifth, both datasets are derived from structured clinical screening contexts and may not represent the full spectrum of image quality encountered in opportunistic or smartphone-based screening.

Finally, this benchmark used a two-layer MLP as the primary classification head. A supplementary ablation comparing MLP versus linear (single-layer) heads was conducted across all three encoders (Supplementary Table S8). All encoders benefited from the nonlinear MLP head, with multiclass macro-F1 improvements of +0.033 for MedSigLIP, +0.030 for RETFound, and +0.016 for EfficientNet-B0. Crucially, the encoder ranking was preserved under both head architectures: MedSigLIP remained the top performer with both linear and MLP heads. These results suggest that while the MLP head provides a uniform performance boost, it does not structurally favour any single encoder. Nevertheless, other adaptation strategies (e.g., parameter-efficient fine-tuning) could yield different relative rankings and remain a direction for future work.

## 5 Conclusion

This benchmark compared the frozen representations of three pretrained encoders—MedSigLIP (medical vision–language), RETFound (retinal self-supervised), and EfficientNet-B0 (ImageNet-supervised)—for diabetic retinopathy classification, using calibration and external generalization as co-primary evaluation axes alongside standard discrimination metrics.

Under internal cross-validation on APTOS 2019, the three encoders were nearly indistinguishable in binary AUC (0.980–0.985), an outcome that could easily lead to the conclusion that encoder choice is immaterial for DR grading. External validation on MESSIDOR-2 overturned this conclusion decisively. MedSigLIP maintained an AUC of 0.915, while RETFound and EfficientNet-B0 fell to 0.697 and 0.745, respectively. The finding that a retina-specific foundation model underperformed an ImageNet baseline under frozen transfer (ΔAUC = −0.051; *p* = 0.016, cluster-robust bootstrap) challenges the assumption that domain-specific pretraining guarantees domain-general representations and underscores the critical importance of evaluating models beyond the development distribution.

The calibration analysis reinforced this message. Temperature scaling successfully reduced expected calibration error on the development set (ECE ≤ 0.022 for all encoders), yet proved largely ineffective under domain shift, with external ECE values ranging from 0.086 to 0.149. In clinical screening programmes where confidence estimates drive triage decisions, this breakdown is consequential: models that appear well calibrated internally may produce systematically overconfident predictions on unseen populations. The Brier score, which jointly captures discrimination and calibration in a single proper scoring rule, proved the most informative summary metric across both datasets and is recommended as a reporting standard for future DR classification studies.

The per-grade analysis exposed a particularly concerning failure mode: all three encoders exhibited catastrophic performance on mild NPDR (grade 1) under domain shift, with RETFound and EfficientNet-B0 achieving F1 = 0.000 and MedSigLIP achieving only 0.153. This finding has direct clinical relevance, as it implies that frozen-encoder classifiers cannot reliably detect the earliest stage of referable pathology in external populations—precisely the stage at which early intervention is most beneficial.

Three actionable recommendations emerge from these findings. First, frozen-encoder benchmarks should treat external validation as the primary evaluation axis; development-set performance alone is insufficient to differentiate encoders or to predict clinical utility. Second, calibration metrics—particularly the Brier score—should be reported alongside AUC as standard practice, because two models with identical AUC can differ substantially in the reliability of their confidence estimates. Third, the resolution confound between MedSigLIP (448 × 448) and the other encoders (224 × 224) means that MedSigLIP’s superior external performance cannot be attributed solely to vision–language pretraining; higher input resolution may contribute to its advantage, particularly for detecting small lesions such as microaneurysms. With this caveat in mind, when frozen transfer is the intended deployment mode, MedSigLIP’s encoder package currently offers the most favourable trade-off between discrimination, calibration, and cross-domain robustness for ophthalmic image analysis.

Future work will expand the framework to additional ophthalmic datasets, incorporate parameter-efficient adaptation methods to assess the complementary dimension of encoder adaptability, explore domain-adaptive calibration strategies that go beyond single-parameter temperature scaling, investigate resolution-matched comparisons when native 224 × 224 variants of medical vision–language encoders become available, and extend the comparison to additional foundation models as they become publicly available.

## Data Availability

The code used for this benchmark (data preprocessing, model definitions, and evaluation scripts) is available at GitHub (https://github.com/opisthion06/diabetic_retinopathy) and archived on Zenodo (DOI: 10.5281/zenodo.19210682).
