# Supplementary material for: How well do frozen foundation models transfer? A calibration-focused benchmark for diabetic retinopathy grading
Source: Front Med (Lausanne). 2026 Apr 16;13:1815982. doi: 10.3389/fmed.2026.1815982 (PMC13128382; doi:10.3389/fmed.2026.1815982)
Supplement: Supplementary file 1 [file Table_1.DOCX]

**SUPPLEMENTARY MATERIAL**

**1. Supplementary Figures and Tables**

**1.1 Supplementary Tables**

**Supplementary Table S1.** Seed variance analysis.

| **Task** | **Encoder** | **AUC μ** | **AUC σ** | **F1 μ** | **F1 σ** | **Brier μ** | **Brier σ** |
| --- | --- | --- | --- | --- | --- | --- | --- |
| Binary | MedSigLIP | 0.9841 | 0.0004 | 0.9266 | 0.0017 | 0.0453 | 0.0006 |
| Binary | RETFound | 0.9831 | 0.0001 | 0.9174 | 0.0014 | 0.0505 | 0.0006 |
| Binary | EffNet-B0 | 0.9794 | 0.0005 | 0.9111 | 0.0029 | 0.0548 | 0.0012 |
| 5-Class | MedSigLIP | — | — | 0.6924 * | 0.0027 | — | — |
| 5-Class | RETFound | — | — | 0.6605 * | 0.0101 | — | — |
| 5-Class | EffNet-B0 | — | — | 0.6295 * | 0.0071 | — | — |

* Macro-F1 reported for 5-class task (AUC and Brier not applicable to multiclass in this format). All values are post-temperature-scaling on the development set.

Mean (μ) and standard deviation (σ) of key metrics across five independent training seeds (13, 17, 23, 29, 31), shown for a representative fold (fold 2). Within-fold AUC SD remained below 0.001 for all encoders, confirming that performance differences reflect encoder properties rather than stochastic MLP initialisation.


**Supplementary Table S2.** Sensitivity analysis: binary referability threshold (grade ≥ 2 vs grade ≥ 1).

*Grade ≥ 2 (primary analysis)*

| **Encoder** | **Set** | **AUC** | **F1** | **ECE↓** | **Brier↓** |
| --- | --- | --- | --- | --- | --- |
| MedSigLIP | Dev | 0.985 ± 0.005 | 0.928 ± 0.015 | 0.014 ± 0.006 | 0.044 ± 0.007 |
| MedSigLIP | Ext | 0.915 ± 0.005 | 0.606 ± 0.024 | 0.109 ± 0.011 | 0.127 ± 0.006 |
| RETFound | Dev | 0.984 ± 0.005 | 0.917 ± 0.014 | 0.018 ± 0.007 | 0.049 ± 0.009 |
| RETFound | Ext | 0.697 ± 0.009 | 0.443 ± 0.046 | 0.086 ± 0.020 | 0.185 ± 0.024 |
| EffNet-B0 | Dev | 0.981 ± 0.007 | 0.916 ± 0.021 | 0.022 ± 0.007 | 0.052 ± 0.011 |
| EffNet-B0 | Ext | 0.745 ± 0.012 | 0.365 ± 0.059 | 0.149 ± 0.023 | 0.182 ± 0.012 |

*Grade ≥ 1 (sensitivity analysis)*

| **Encoder** | **Set** | **AUC** | **F1** | **ECE↓** | **Brier↓** |
| --- | --- | --- | --- | --- | --- |
| MedSigLIP | Dev | 1.000 ± 0.000 | 0.990 ± 0.002 | 0.006 ± 0.002 | 0.008 ± 0.002 |
| MedSigLIP | Ext | 0.867 ± 0.012 | 0.701 ± 0.031 | 0.157 ± 0.004 | 0.174 ± 0.006 |
| RETFound | Dev | 0.995 ± 0.001 | 0.974 ± 0.005 | 0.010 ± 0.004 | 0.021 ± 0.003 |
| RETFound | Ext | 0.654 ± 0.007 | 0.343 ± 0.077 | 0.229 ± 0.031 | 0.282 ± 0.015 |
| EffNet-B0 | Dev | 0.996 ± 0.000 | 0.975 ± 0.006 | 0.009 ± 0.005 | 0.019 ± 0.003 |
| EffNet-B0 | Ext | 0.675 ± 0.007 | 0.359 ± 0.050 | 0.278 ± 0.013 | 0.302 ± 0.008 |

Grade ≥ 2: grades 0–1 = non-referable, grades 2–4 = referable (ICDR standard). Grade ≥ 1: grade 0 = non-referable, grades 1–4 = referable (alternative threshold). All values post-temperature scaling. Dev = out-of-fold on APTOS; Ext = zero-tuning on MESSIDOR-2.

**Supplementary Table S3.** External fold-ensemble performance on MESSIDOR-2.

*Binary referable DR.*

| **Encoder** | **Pre AUC** | **Post AUC** | **Pre F1** | **Post F1** | **Pre ECE** | **Post ECE** | **Pre Brier** | **Post Brier** |
| --- | --- | --- | --- | --- | --- | --- | --- | --- |
| MedSigLIP | 0.914 | 0.915 | 0.611 | 0.611 | 0.108 | 0.105 | 0.127 | 0.126 |
| RETFound | 0.698 | 0.697 | 0.469 | 0.467 | 0.051 | 0.052 | 0.168 | 0.169 |
| EffNet-B0 | 0.751 | 0.748 | 0.356 | 0.356 | 0.155 | 0.143 | 0.181 | 0.177 |

*Five-class severity grading.*

| **Encoder** | **Pre Mac-F1** | **Post Mac-F1** | **Pre QWK** | **Post QWK** | **Pre Bal-Acc** | **Post Bal-Acc** | **Pre ECE** | **Post ECE** |
| --- | --- | --- | --- | --- | --- | --- | --- | --- |
| MedSigLIP | 0.447 | 0.443 | 0.714 | 0.714 | 0.414 | 0.413 | 0.216 | 0.214 |
| RETFound | 0.248 | 0.248 | 0.352 | 0.350 | 0.301 | 0.301 | 0.168 | 0.190 |
| EffNet-B0 | 0.302 | 0.302 | 0.352 | 0.350 | 0.313 | 0.315 | 0.271 | 0.260 |

Soft-probability predictions from all five CV folds were averaged before thresholding, representing a practical deployment scenario. Pre = before temperature scaling; Post = after temperature scaling. AUC is not applicable to the multiclass fold-ensemble in panel B. Mac-F1 = macro-averaged F1; QWK = quadratic weighted kappa; Bal-Acc = balanced accuracy; ECE = expected calibration error (10 bins).


**Supplementary Table S4.** Complete pairwise statistical comparisons between frozen encoders.

**DEV — Binary**

| **Comparison** | **Metric** | **Test** | **Δ** | **95% CI** | **p (BH)** |  |
| --- | --- | --- | --- | --- | --- | --- |
| MedSigLIP vs RETFound | AUC | DeLong | +0.0015 | — | 0.281 |  |
| MedSigLIP vs RETFound | Correctness | McNemar | +0.0082 | — | 0.062 |  |
| MedSigLIP vs RETFound | ECE | Boot | −0.0010 | [−0.007, +0.005] | 0.766 |  |
| MedSigLIP vs RETFound | Brier | Boot | −0.0053 | [−0.009, −0.001] | 0.030 | † |
| MedSigLIP vs EffNet-B0 | AUC | DeLong | +0.0048 | — | 0.003 | † |
| MedSigLIP vs EffNet-B0 | Correctness | McNemar | +0.0090 | — | 0.062 |  |
| MedSigLIP vs EffNet-B0 | ECE | Boot | −0.0045 | [−0.011, +0.003] | 0.321 |  |
| MedSigLIP vs EffNet-B0 | Brier | Boot | −0.0083 | [−0.012, −0.004] | 0.006 | † |
| RETFound vs EffNet-B0 | AUC | DeLong | +0.0033 | — | 0.043 | † |
| RETFound vs EffNet-B0 | Correctness | McNemar | +0.0008 | — | 0.894 |  |
| RETFound vs EffNet-B0 | ECE | Boot | −0.0035 | [−0.011, +0.004] | 0.422 |  |
| RETFound vs EffNet-B0 | Brier | Boot | −0.0031 | [−0.007, +0.001] | 0.304 |  |

**DEV — Multiclass**

| **Comparison** | **Metric** | **Test** | **Δ** | **95% CI** | **p (BH)** |  |
| --- | --- | --- | --- | --- | --- | --- |
| MedSigLIP vs RETFound | Mac-F1 | Boot | +0.0371 | [+0.014, +0.059] | 0.002 | † |
| MedSigLIP vs RETFound | QWK | Boot | +0.0216 | [+0.011, +0.033] | 0.002 | † |
| MedSigLIP vs RETFound | ECE | Boot | −0.0062 | [−0.019, +0.006] | 0.402 |  |
| MedSigLIP vs RETFound | Brier | Boot | −0.0428 | [−0.055, −0.031] | 0.002 | † |
| MedSigLIP vs EffNet-B0 | Mac-F1 | Boot | +0.0628 | [+0.041, +0.086] | 0.002 | † |
| MedSigLIP vs EffNet-B0 | QWK | Boot | +0.0489 | [+0.036, +0.061] | 0.002 | † |
| MedSigLIP vs EffNet-B0 | ECE | Boot | +0.0037 | [−0.009, +0.017] | 0.576 |  |
| MedSigLIP vs EffNet-B0 | Brier | Boot | −0.0576 | [−0.069, −0.047] | 0.002 | † |
| RETFound vs EffNet-B0 | Mac-F1 | Boot | +0.0257 | [+0.004, +0.048] | 0.035 | † |
| RETFound vs EffNet-B0 | QWK | Boot | +0.0272 | [+0.014, +0.040] | 0.002 | † |
| RETFound vs EffNet-B0 | ECE | Boot | +0.0099 | [−0.003, +0.022] | 0.161 |  |
| RETFound vs EffNet-B0 | Brier | Boot | −0.0148 | [−0.026, −0.005] | 0.003 | † |

**EXT — Binary**

| **Comparison** | **Metric** | **Test** | **Δ** | **95% CI** | **p (BH)** |  |
| --- | --- | --- | --- | --- | --- | --- |
| MedSigLIP vs RETFound | AUC | DeLongᵃ | +0.2175 | — | <0.001 | † |
| MedSigLIP vs RETFound | AUC | Clust. Boot | +0.2185 | [+0.180, +0.258] | <0.001 | † |
| MedSigLIP vs RETFound | Correctness | McNemar | +0.0780 | — | <0.001 | † |
| MedSigLIP vs RETFound | ECE | Clust. Boot | +0.0503 | [+0.026, +0.073] | <0.001 | † |
| MedSigLIP vs RETFound | Brier | Clust. Boot | −0.0433 | [−0.056, −0.030] | <0.001 | † |
| MedSigLIP vs EffNet-B0 | AUC | DeLongᵃ | +0.1671 | — | <0.001 | † |
| MedSigLIP vs EffNet-B0 | AUC | Clust. Boot | +0.1674 | [+0.140, +0.196] | <0.001 | † |
| MedSigLIP vs EffNet-B0 | Correctness | McNemar | +0.0642 | — | <0.001 | † |
| MedSigLIP vs EffNet-B0 | ECE | Clust. Boot | −0.0408 | [−0.057, −0.026] | <0.001 | † |
| MedSigLIP vs EffNet-B0 | Brier | Clust. Boot | −0.0532 | [−0.064, −0.043] | <0.001 | † |
| RETFound vs EffNet-B0 | AUC | DeLongᵃ | −0.0504 | — | 0.003 | † |
| RETFound vs EffNet-B0 | AUC | Clust. Boot | −0.0511 | [−0.091, −0.013] | 0.016 | † |
| RETFound vs EffNet-B0 | Correctness | McNemar | −0.0138 | — | 0.131 |  |
| RETFound vs EffNet-B0 | ECE | Clust. Boot | −0.0911 | [−0.114, −0.067] | <0.001 | † |
| RETFound vs EffNet-B0 | Brier | Clust. Boot | −0.0098 | [−0.023, +0.003] | 0.140 |  |

**EXT — Multiclass**

| **Comparison** | **Metric** | **Test** | **Δ** | **95% CI** | **p (BH)** |  |
| --- | --- | --- | --- | --- | --- | --- |
| MedSigLIP vs RETFound | Mac-F1 | Clust. Boot | +0.1932 | [+0.142, +0.241] | <0.001 | † |
| MedSigLIP vs RETFound | QWK | Clust. Boot | +0.3645 | [+0.307, +0.423] | <0.001 | † |
| MedSigLIP vs RETFound | ECE | Clust. Boot | +0.0223 | [+0.001, +0.043] | 0.044 | † |
| MedSigLIP vs RETFound | Brier | Clust. Boot | −0.1320 | [−0.155, −0.107] | <0.001 | † |
| MedSigLIP vs EffNet-B0 | Mac-F1 | Clust. Boot | +0.1380 | [+0.082, +0.191] | <0.001 | † |
| MedSigLIP vs EffNet-B0 | QWK | Clust. Boot | +0.3656 | [+0.308, +0.423] | <0.001 | † |
| MedSigLIP vs EffNet-B0 | ECE | Clust. Boot | −0.0581 | [−0.076, −0.041] | <0.001 | † |
| MedSigLIP vs EffNet-B0 | Brier | Clust. Boot | −0.1443 | [−0.167, −0.123] | <0.001 | † |
| RETFound vs EffNet-B0 | Mac-F1 | Clust. Boot | −0.0553 | [−0.105, −0.004] | 0.040 | † |
| RETFound vs EffNet-B0 | QWK | Clust. Boot | +0.0011 | [−0.061, +0.069] | 0.969 |  |
| RETFound vs EffNet-B0 | ECE | Clust. Boot | −0.0804 | [−0.098, −0.063] | <0.001 | † |
| RETFound vs EffNet-B0 | Brier | Clust. Boot | −0.0123 | [−0.034, +0.008] | 0.249 |  |

Δ = model A minus model B. † Significant at p < 0.05 after Benjamini–Hochberg correction within each dataset–task–test group. Boot, paired bootstrap (1,000 iterations, image-level). Clust. Boot, patient-level cluster bootstrap (2,000 iterations).

ᵃ DeLong test assumes image-level independence; reported on the external set alongside cluster-robust results for transparency. CI not applicable (—) for DeLong and McNemar.


**Supplementary Table S5.** Encoder profiles: architecture, pretraining paradigm, parameter count, native input resolution, and batch inference throughput.

| **Encoder** | **Architecture** | **Pretraining** | **Parameters** | **Resolution** | **Throughput** |
| --- | --- | --- | --- | --- | --- |
| MedSigLIP | ViT-B/16 | Medical VL (33 M pairs) | 878 M | 448 × 448 | 12.5 img/s |
| RETFound | ViT-L/16 | Retinal MAE (1.6 M images) | 303 M | 224 × 224 | 106.8 img/s |
| EffNet-B0 | CNN (compound) | ImageNet-1K (supervised) | 4 M | 224 × 224 | 1 211.6 img/s |

Throughput measured on a single NVIDIA T4 GPU at batch size 16 with mixed-precision inference. VL = vision–language; MAE = masked autoencoder. Parameters include all encoder weights (frozen during training). Throughput reflects embedding extraction only, excluding MLP head inference. MedSigLIP throughput reflects the higher computational cost of the 448 × 448 input resolution and the vision–language dual-encoder architecture (text encoder discarded at inference).


**Supplementary Table S6.** Per-grade precision, recall, and F1 for five-class ICDR severity grading on development (Dev) and external (Ext) sets.

| **Encoder** | **Set** | **Grade** | **Precision** | **Recall** | **F1** | **n** | **95% CI** |
| --- | --- | --- | --- | --- | --- | --- | --- |
| MedSigLIP | Dev | 0 | 0.994 | 0.985 | 0.990 | 1805 | — |
| MedSigLIP | Dev | 1 | 0.612 | 0.703 | 0.654 | 370 | — |
| MedSigLIP | Dev | 2 | 0.786 | 0.741 | 0.763 | 999 | — |
| MedSigLIP | Dev | 3 | 0.476 | 0.513 | 0.494 | 193 | — |
| MedSigLIP | Dev | 4 | 0.633 | 0.644 | 0.639 | 295 | — |
| MedSigLIP | Ext | 0 | 0.775 | 0.978 | 0.865 | 1017 | — |
| MedSigLIP | Ext | 1 | 0.154 | 0.152 | 0.153 | 270 | — |
| MedSigLIP | Ext | 2 | 0.539 | 0.219 | 0.311 | 347 | — |
| MedSigLIP | Ext | 3 | 0.556 | 0.200 | 0.294 | 75 | [0.187, 0.404] |
| MedSigLIP | Ext | 4 | 0.692 | 0.514 | 0.590 | 35 | [0.436, 0.716] |
| RETFound | Dev | 0 | 0.971 | 0.970 | 0.970 | 1805 | — |
| RETFound | Dev | 1 | 0.523 | 0.700 | 0.599 | 370 | — |
| RETFound | Dev | 2 | 0.772 | 0.704 | 0.736 | 999 | — |
| RETFound | Dev | 3 | 0.409 | 0.513 | 0.455 | 193 | — |
| RETFound | Dev | 4 | 0.714 | 0.508 | 0.594 | 295 | — |
| RETFound | Ext | 0 | 0.622 | 0.934 | 0.747 | 1017 | — |
| RETFound | Ext | 1 | 0.000 | 0.000 | 0.000 | 270 | — |
| RETFound | Ext | 2 | 0.620 | 0.089 | 0.156 | 347 | — |
| RETFound | Ext | 3 | 0.205 | 0.453 | 0.282 | 75 | [0.223, 0.344] |
| RETFound | Ext | 4 | 1.000 | 0.029 | 0.056 | 35 | [0.000, 0.158] |
| EffNet-B0 | Dev | 0 | 0.975 | 0.968 | 0.972 | 1805 | — |
| EffNet-B0 | Dev | 1 | 0.571 | 0.686 | 0.623 | 370 | — |
| EffNet-B0 | Dev | 2 | 0.740 | 0.672 | 0.704 | 999 | — |
| EffNet-B0 | Dev | 3 | 0.392 | 0.508 | 0.442 | 193 | — |
| EffNet-B0 | Dev | 4 | 0.509 | 0.461 | 0.484 | 295 | — |
| EffNet-B0 | Ext | 0 | 0.616 | 0.984 | 0.757 | 1017 | — |
| EffNet-B0 | Ext | 1 | 0.000 | 0.000 | 0.000 | 270 | — |
| EffNet-B0 | Ext | 2 | 0.356 | 0.046 | 0.082 | 347 | — |
| EffNet-B0 | Ext | 3 | 0.568 | 0.280 | 0.375 | 75 | [0.250, 0.471] |
| EffNet-B0 | Ext | 4 | 0.333 | 0.286 | 0.308 | 35 | [0.167, 0.429] |

Dev = out-of-fold on APTOS 2019; Ext = zero-tuning on MESSIDOR-2. n = number of images per grade. Values rounded to three decimal places. All values are post-temperature-scaling. Bootstrapped 95% confidence intervals (2,000 resamples) are reported for external grades 3 and 4, where sample sizes are small (n = 75 and n = 35, respectively). Grade 1 F1 = 0.000 for RETFound and EfficientNet-B0 on external set.


**Table S7.** Five-class temperature scaling parameters across folds.

| **Encoder** | **Fold 0** | **Fold 1** | **Fold 2** | **Fold 3** | **Fold 4** | **Mean ± SD** |
| --- | --- | --- | --- | --- | --- | --- |
| MedSigLIP | 1.115 | 1.080 | 1.101 | 0.849 | 1.010 | 1.031 ± 0.109 |
| RETFound | 0.866 | 0.893 | 0.951 | 1.010 | 0.865 | 0.917 ± 0.063 |
| EffNet-B0 | 1.255 | 1.172 | 0.782 | 1.102 | 1.320 | 1.126 ± 0.209 |

Temperature values are seed-averaged within each fold (5 seeds per fold). T > 1 indicates softening (reducing overconfidence); T < 1 indicates sharpening. RETFound exhibited the most stable temperatures across folds (SD = 0.063), while EfficientNet-B0 showed the highest variability (SD = 0.209), with fold 2 producing a notably low T = 0.782.

**Supplementary Table S8.** Linear vs MLP head ablation on the APTOS development set.

*Binary referable DR classification*

| **Encoder** | **Head** | **AUC** | **F1** | **ECE↓** | **Brier↓** |
| --- | --- | --- | --- | --- | --- |
| MedSigLIP | Linear | 0.982 ± 0.006 | 0.922 ± 0.014 | 0.014 ± 0.004 | 0.049 ± 0.008 |
| MedSigLIP | MLP | 0.985 ± 0.005 | 0.928 ± 0.015 | 0.014 ± 0.006 | 0.044 ± 0.007 |
| RETFound | Linear | 0.980 ± 0.006 | 0.909 ± 0.013 | 0.025 ± 0.012 | 0.056 ± 0.009 |
| RETFound | MLP | 0.984 ± 0.005 | 0.917 ± 0.014 | 0.018 ± 0.007 | 0.049 ± 0.009 |
| EffNet-B0 | Linear | 0.976 ± 0.009 | 0.908 ± 0.026 | 0.026 ± 0.009 | 0.057 ± 0.013 |
| EffNet-B0 | MLP | 0.981 ± 0.007 | 0.916 ± 0.021 | 0.022 ± 0.007 | 0.052 ± 0.011 |

*Five-class severity grading*

| **Encoder** | **Head** | **Mac-F1** | **QWK** | **Bal-Acc** | **ECE↓** | **Brier↓** |
| --- | --- | --- | --- | --- | --- | --- |
| MedSigLIP | Linear | 0.675 ± 0.007 | 0.896 ± 0.004 | 0.706 ± 0.009 | 0.047 ± 0.021 | 0.263 ± 0.010 |
| MedSigLIP | MLP | 0.708 ± 0.018 | 0.906 ± 0.008 | 0.717 ± 0.016 | 0.029 ± 0.014 | 0.232 ± 0.012 |
| RETFound | Linear | 0.641 ± 0.016 | 0.858 ± 0.021 | 0.653 ± 0.023 | 0.095 ± 0.017 | 0.320 ± 0.011 |
| RETFound | MLP | 0.670 ± 0.012 | 0.884 ± 0.016 | 0.679 ± 0.018 | 0.039 ± 0.009 | 0.275 ± 0.010 |
| EffNet-B0 | Linear | 0.628 ± 0.006 | 0.847 ± 0.019 | 0.652 ± 0.011 | 0.049 ± 0.007 | 0.308 ± 0.010 |
| EffNet-B0 | MLP | 0.644 ± 0.012 | 0.857 ± 0.016 | 0.659 ± 0.012 | 0.032 ± 0.007 | 0.289 ± 0.006 |

Linear head: single-seed (seed 13), 5-fold mean ± SD. MLP head: 5-seed averaged, 5-fold mean ± SD (from main analysis). All values on development set, post-temperature scaling for MLP, pre-temperature scaling for linear.

**1.2 Supplementary Figures**


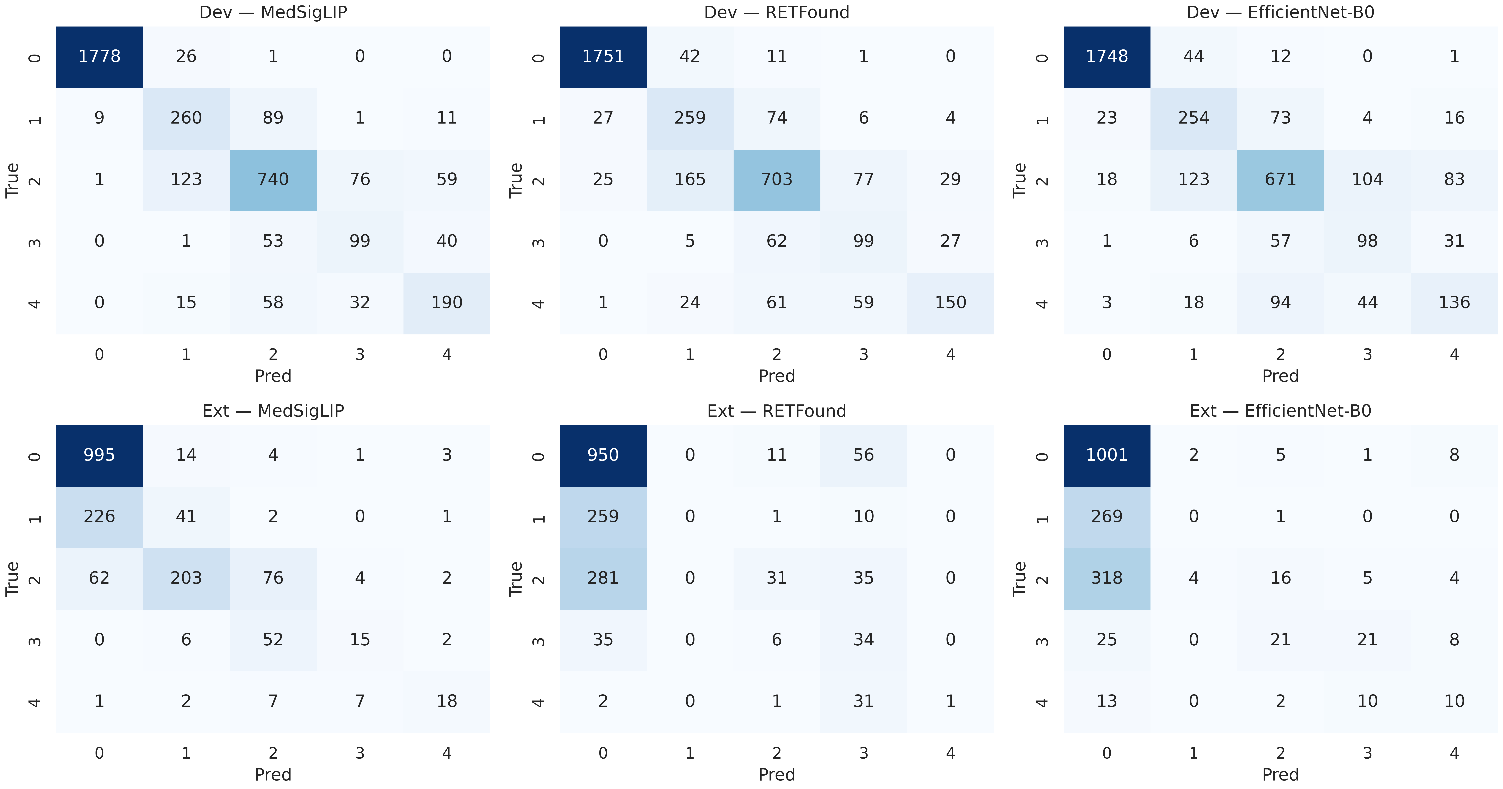


**Supplementary Figure S1.** Confusion matrices for five-class ICDR severity grading (grades 0–4). Upper row: development set (Dev; out-of-fold on APTOS 2019); lower row: external set (Ext; MESSIDOR-2). Each column corresponds to one encoder. On the external set, off-diagonal mass is concentrated toward the grade-0 **column** (predicted), indicating systematic misclassification of pathological grades as non-referable.


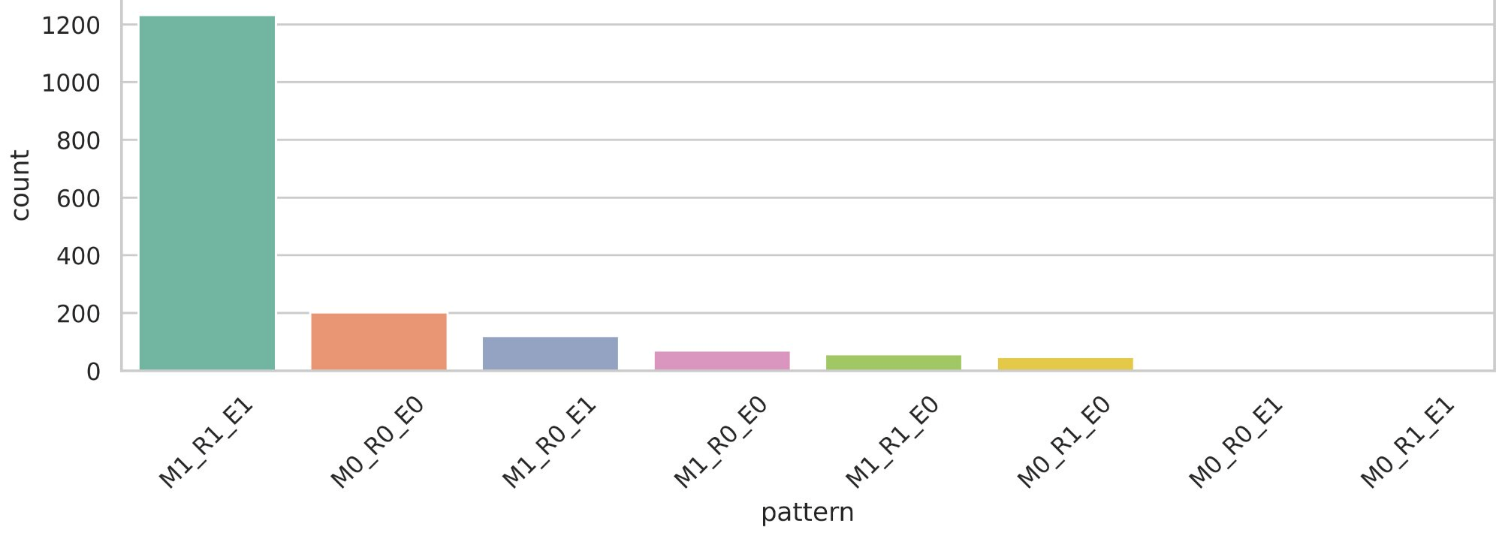


**Supplementary Figure S2.** Error overlap patterns for binary referable DR classification on MESSIDOR-2. Each bar represents a unique correct (1) / incorrect (0) pattern across MedSigLIP (M), RETFound (R), and EfficientNet-B0 (E); e.g., M1_R0_E0 denotes MedSigLIP correct while both others are incorrect. The dominant M1_R1_E1 pattern (all correct) is followed by M0_R0_E0 (all incorrect). The M1_R0_E0 pattern substantially outnumbers the reverse patterns, confirming MedSigLIP's unique error-correction capacity.


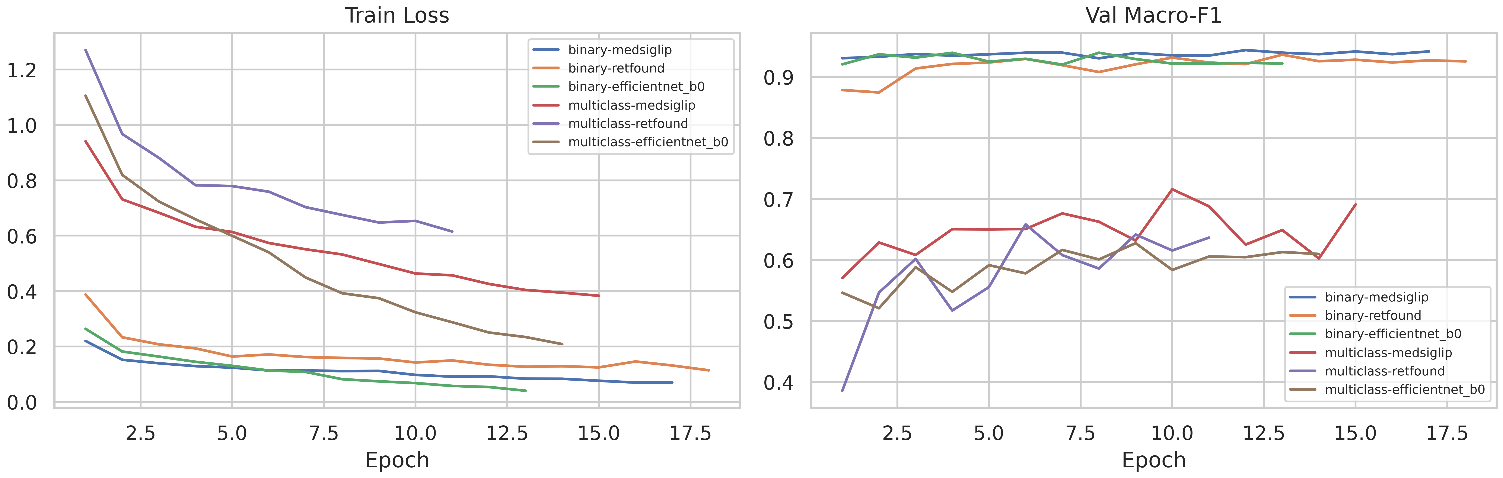


**Supplementary Figure S3.** Training dynamics for a representative configuration (fold 0, seed 13). Left: weighted cross-entropy training loss versus epoch. Right: validation macro-averaged F1 (macro-F1) versus epoch. Early stopping patience = 5 epochs. MedSigLIP embeddings yield a lower terminal training loss across both binary referable diabetic retinopathy (DR) and five-class International Clinical Diabetic Retinopathy (ICDR) severity grading tasks.


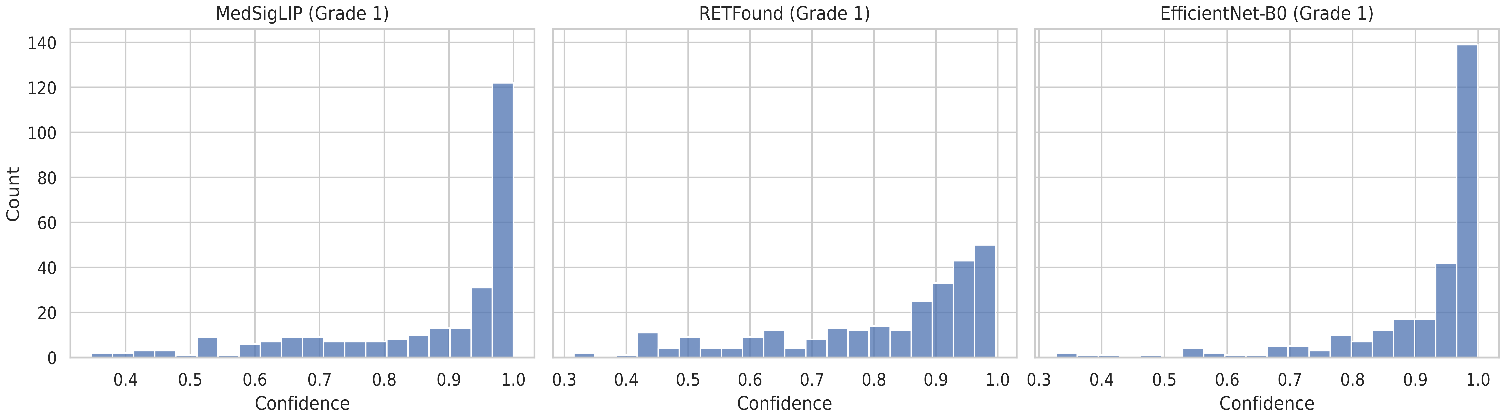


**Supplementary Figure S4.** Confidence distributions for true grade-1 (mild non-proliferative DR) images on the external set (MESSIDOR-2; n = 270). Histograms show the maximum predicted class probability (i.e., model confidence in its top prediction) for each encoder. RETFound and EfficientNet-B0 concentrate confidence near 1.0, reflecting overconfident misclassification as grade 0. MedSigLIP displays a wider confidence spread, consistent with its non-zero albeit low grade-1 recall (F1 = 0.153).
